# Randomized controlled trial of a group peer mentoring model for U.S. academic medicine research faculty

**DOI:** 10.1017/cts.2023.589

**Published:** 2023-08-22

**Authors:** Linda H. Pololi, Arthur T. Evans, Mark Brimhall-Vargas, Janet T. Civian, Lisa A. Cooper, Brian K. Gibbs, Kacy Ninteau, Vasilia Vasiliou, Robert T. Brennan

**Affiliations:** 1 National Initiative on Gender, Culture and Leadership in Medicine: C-Change, Institute for Economic and Racial Equity, The Heller School for Social Policy and Management, Brandeis University, Waltham, MA, USA; 2 Division of Hospital Medicine, Weill Cornell Medical College, New York, NY, USA; 3 Racial Equity and Social Justice, Fenway Health, Boston, MA, USA; 4 Brandeis University, Waltham, MA, USA; 5 Johns Hopkins University School of Medicine and Bloomberg School of Public Health, Baltimore, MD, USA; 6 UMass Memorial Health, Worcester, MA, USA

**Keywords:** Physician investigators, faculty, academic medicine, mentoring, diversity, translational research, culture, career advancement, relationships, peer mentoring, PhD, race, ethnicity, burnout, self efficacy, research faculty

## Abstract

**Introduction:**

Midcareer is a critical transition point for biomedical research faculty and a common dropout point from an NIH-funded career. We report a study to assess the efficacy of a group peer mentoring program for diverse biomedical researchers in academic medicine, seeking to improve vitality, career advancement, and cross-cultural competence.

**Methods:**

We conducted a stratified randomized controlled trial with a waitlist control group involving 40 purposefully diverse early midcareer research faculty from 16 states who had a first-time NIH R01 (or equivalent) award, a K training grant, or a similar major grant. The yearlong intervention (2 to 3 days quarterly) consisted of facilitated, structured, group peer mentoring. Main study aims were to enhance faculty vitality, self-efficacy in achieving research success, career advancement, mentoring others, and cultural awareness and appreciation of diversity in the workplace.

**Results:**

Compared to the control group, the intervention group’s increased vitality did not reach statistical significance (*P* = 0.20), but perceived change in vitality was 1.47 standard deviations higher (*D* = 1.47, *P* = 0.03). Self-efficacy for career advancement was higher in the intervention group (*D* = 0.41, *P* = 0.05) as was self-efficacy for research (*D* = 0.57, *P* = 0.02). The intervention group also valued diversity higher (*D* = 0.46, *P* = 0.02), had higher cognitive empathy (*D* = 0.85, *P* = 0.03), higher anti-sexism/racism skills (*D* = 0.71, *P* = 0.01), and higher self-efficacy in mentoring others (*D* = 1.14, *P* = 0.007).

**Conclusions:**

The mentoring intervention resulted in meaningful change in important dimensions and skills among a national sample of diverse early midcareer biomedical faculty. This mentoring program holds promise for addressing the urgencies of sustaining faculty vitality and cross-cultural competence.

## Introduction

Maintaining the strength and competitiveness of U.S. medical science depends on maintaining a strong and appropriately diverse science workforce. There are high levels of research faculty attrition, with about 40% loss from NIH funding after completing an initial R01 research project grant [1–4]. These faculty are mostly midcareer.

Furthermore, there remains a striking lack of diversity in this biomedical research workforce [1]. Although large numbers of women and historically excluded racial and ethnic group members have completed M.D. and Ph.D. training, these faculty groups have experienced more barriers to career advancement and more attrition from academic biomedical research careers [2,5,6].

Mentoring is widely recommended in medical schools as a means to revitalize academic medicine and decrease faculty turnover [7–9]; yet only a third of medical school faculty reports good mentoring [10]. Improved mentoring of early midcareer researchers, including women and persons from underrepresented racial and ethnic groups, has been identified as an important potential avenue for addressing these observations and for treating the reported current low levels of vitality and high burnout in biomedical faculty [11–13]. Dyadic (one-on-one) mentoring has been the predominant model, but accumulated evidence suggests that this model is insufficient to meet existing needs [10]. There is increasing recognition of the need for improved forms of mentoring that can be scaled up to better meet the needs of the national biomedical science workforce.

In the randomized controlled study reported here, our objective was to test the efficacy of a group peer mentoring program among academic early midcareer physicians and scientists engaged in biomedical research, including women and persons from historically excluded and underrepresented racial and ethnic groups. The mentoring program, the C-Change (for culture change) Mentoring & Leadership Institute (“Institute”), has been implemented and refined among physicians and scientists over the past decade [14,15]. We hypothesized that intervention participants would demonstrate enhanced vitality and career advancement, and greater cultural awareness and appreciation of diversity, indicating cross-cultural competence.

## Methods

### Recruitment of study participants

In 2020 from across the U.S., we recruited 99 eligible early midcareer research faculty and randomly allocated 40 to either intervention or control group (1:1 allocation), stratified by gender, degree, and race and ethnicity. Inclusion criteria were: 1) appointment for 3–14 years at a U.S. medical school or teaching hospital; 2) associate professor or at least two years at rank of assistant professor (or equivalent); and 3) demonstrated research that included a current or recent first-time NIH R01 or R01-equivalent award; R21 or R34 award; HRSA, ARHQ, or other federal agency major grant; K training grant; or recent major foundation or professional organization grant. We excluded those with more than one R01-equivalent award so as to focus the investigation on faculty most vulnerable to attrition given the high attrition rate from federal funding of first-time R01 awardees [3].

To obtain the sampling frame, NIH RePORTER was searched for all awardees receiving qualifying grants from 2013 to 2019. Because the study design called for 50% participation by persons from underrepresented racial and ethnic groups as defined by NIH [16] (Black/African-American, Hispanic/Latinx, Native American, Alaska Native, and Pacific Islander), additional methods were used for focused recruitment. First, grant titles using words associated with diversity concepts (e.g. disparities) were flagged. Second, photos of researchers were located through Internet searches and scored for likely race and ethnicity using Kairos online facial recognition software (www.kairos.com) [17]. Third, a list of common Latinx surnames was used to identify possible Latinx researchers. Deans and others with responsibility for diversity at medical institutions were contacted to identify and alert eligible faculty members of the opportunity to participate in the Institute. Those identified as possibly belonging to these underrepresented groups received additional customized emails.

**Figure 1.**
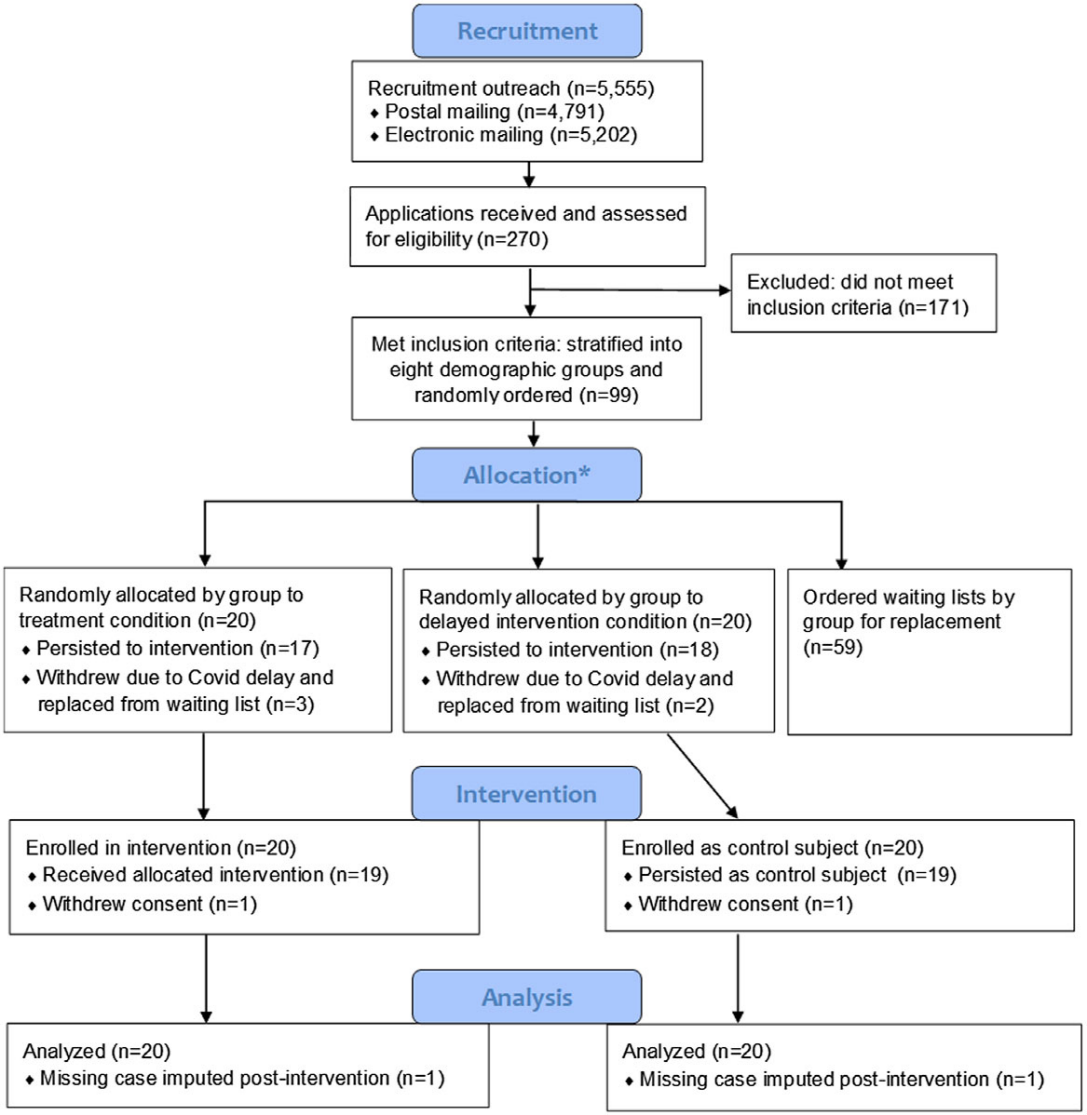
Subject recruitment and inclusion. *The 99 applicants who met the inclusion criteria were stratified into eight groups by underrepresented or non-underrepresented in medicine, male or female, and degree (MD or MD/PhD versus PhD), randomly ordered within each group, and then assigned to the initial intervention (treatment) or waitlist control (delayed intervention). Those remaining in each group were used as ordered replacements if an invited applicant from the group withdrew.

The sources consulted did not provide needed information to apply inclusion/exclusion criteria, so recruiting covered all potential subjects located via our search criteria for whom we could find contact information. Hard copy invitations to apply to the Institute were mailed to 4,791 individuals in the recruitment pool, and emails were sent to 5,202 individuals, with most receiving both methods of communication (4,438). Those wishing to participate completed applications that included self-reports of the inclusion criteria for the study and a current CV. Additionally, applicants consented that should they be selected, they would participate in one of two annual Institutes. Of 270 applications received, 99 met all inclusion criteria (see Fig. 1). The size of the intervention group (20), as well as control (20), which would be treated after one year, was based on the maximum size that could be accommodated within the format of the Institute.

### Design

The study used a stratified random design with a waitlist control (delayed intervention) group. Subjects were randomized to participate in the initial yearlong intervention group or a waitlist control group that would start the Institute 12 months later. In our parallel randomized group design, any effects of the SARS-2-CoV pandemic were well controlled because data were collected at the same time for both the intervention and waitlist control groups.

Those eligible were stratified by three binary characteristics: non-underrepresented vs. underrepresented in medicine, male vs. female, and M.D. (or MD/PhD) vs. Ph.D. degree. The priority was to maintain a 50:50 balance within the first two characteristics: race and ethnicity, and gender. Actual identification of race and ethnicity was obtained from self-report in applications, not the methods used for enhanced recruitment. Within each of the resulting cells, the respondents were put on randomly ordered lists in Excel v.2019 (Microsoft, Redmond, WA) to be assigned to a) the initial treatment group of 20, b) the delayed intervention group of 20, or c) a waiting list used in randomized order to replace any losses, such as failure to accept the offer or schedule modifications due to COVID-19 (Fig. 1).

Participants in both the treatment and control groups were assessed with an online survey prior to the commencement of the first Institute at baseline in November 2020, and again one year later at follow-up, after completion of the first Institute. Brandeis University Human Subjects Protection IRB approved this study IRB #19127R-E.

### Intervention

The intervention – the Institute – entailed a facilitated group process characterized by nonhierarchical peer relationships, empowerment, self-direction, and reflection. Theoretical foundations for the Institute were adult learning theory [18–20], Rogerian psychological principles [21], praxis [22], development of personal awareness [23], cognitive empathy [24], and self-determination theory [25].

**Figure 2.**
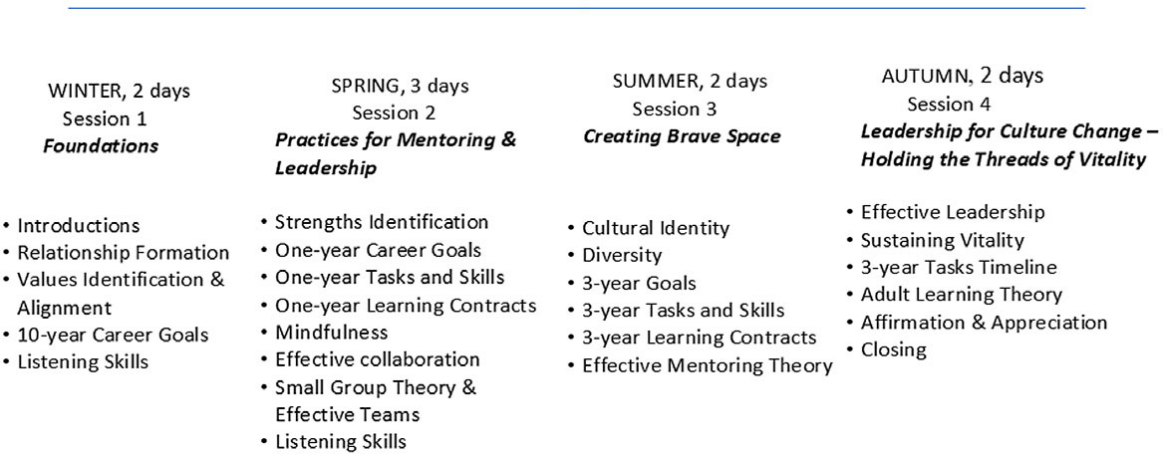
C-Change Mentoring & Leadership Institute curriculum content.

Participants, simultaneously holding the roles of both mentor and protégé, worked closely with peers from multiple institutions during a yearlong course that convened in person quarterly for two- or three-day sessions. During each intensive session, participants engaged in a highly structured process of career planning and learning skills in key areas for career advancement. The sessions employed experiential, cognitive, and affective learning methods. The curriculum addressed knowledge and skills essential for leaders and advancement in academic medicine, and for effective mentoring. Curricular content focused on relationship formation, identification of personal core values and their alignment with career and personal goals, listening skills, identification of strengths, mindfulness, effective collaboration and teamwork, appreciation of diversity and cultural self-identity, effective mentoring and leadership models, and sustaining vitality (Fig. 2). Each participant was guided through the steps of formulating an explicit written personal career plan that included short- and long-term goals and identification of the ways to accomplish the goals [26].

Each curricular content area was taught in a variety of ways to include the differently preferred ways by which individuals learn including both small group and a large group exchange of ideas. Often, this was followed by individual reflective writing on the learning experience and a debriefing large group dialog where participants were invited to articulate their own learning and thus also learn about the learning of other participants. Relevant key articles were provided for each content area, usually to be read after the session. The facilitators maintained the agenda and a safe space for dialog within large group events. Participants mentored each other as peers during frequent dedicated small group sessions of two or three participants. The facilitators brought formal attention to the Institute’s learning culture and stimulated communication within the group. Due to COVID-19-induced travel restrictions, the first two sessions of the initial intervention cohort were conducted by virtual conferencing.

### Outcome measures

Table 1 shows details of each of the scales used.

**Table 1.** C-Change survey domain descriptions, response scales, reliability, and estimated means by group and timepoint^a^ for 40 research faculty participating in the C-Change group peer mentoring study^b^

Domain description (number of items)	Scale	Estimated Cronbach alpha	Intervention group mean (SD) time^0^	Intervention group mean (SD) time^1^	Control group mean (SD) time^0^	Control group mean (SD) time^1^
**Vitality (4)** *Being energized by work*	1–6	0.89	4.8 (0.9)	5.1 (0.9)	4.8 (1.0)	4.8 (0.7)
**Self-assessed change in vitality (4)** *Perception of current vitality compared to one year ago*	1–7	0.93	4.0 (1.2)	5.4 (1.4)	4.6 (1.3)	4.4 (0.9)
**Self-efficacy: career advancement (4)** *Self-confidence in ability to advance in career*	1–7	0.83	5.2 (1.2)	5.8 (1.2)	5.0 (1.6)	5.2 (1.1)
**Self-efficacy: research (4)** *Self-confidence in ability to be successful in research*	1–6	0.87	3.6 (1.0)	4.3 (1.0)	4.0 (1.2)	4.0 (1.0)
**Valuing diversity: attitudes and recruitment (9)** *Extent of valuing diversity in work teams, recruitment, and advancement*	1–6	0.89	5.1 (0.9)	5.4 (0.7)	4.9 (0.9)	4.9 (0.8)
**Cognitive empathy (6)** *Ability to identify concerns and discomfort in others*	1–7	0.89	5.2 (1.0)	5.6 (0.8)	5.1 (1.2)	4.7 (1.5)
**Anti-sexism/anti-racism skills (4)** *Ability to identify and respond to gender, race, and ethnicity inequity*	1–7	0.84	5.3 (0.9)	5.9 (0.7)	5.2 (1.4)	5.2 (1.1)
**Self-efficacy: mentoring others (7)** *Self-confidence in ability to effectively mentor others*	1–6	0.91	4.4 (0.7)	5.1 (0.7)	4.2 (1.2)	4.2 (1.1)

a Timepoints Time^0^ and Time^1^ represent measurement prior to and following the intervention period.

b Means calculated using all domain items. Imputation conducted at the item level in cases where respondent answered at least 50% of the domain items.

#### Change in vitality and self-efficacy

To test for group differences in one of our primary outcomes, vitality, we included two scales. One included four items using a six-point ordinal frequency response scale ranging from “never” to “very frequently,” assessing satisfaction, energy, and meaningful work, derived from the validated C-Change Faculty Survey [11,12]. The second vitality scale used the same four items, but asked participants to assess their perceived change in each aspect of vitality over a twelve-month period (i.e., “compared to a year ago”) with responses on a seven-point Likert agreement scale from “very strongly disagree” to “very strongly agree.”

For our second primary outcome, self-efficacy in career advancement, we used two scales. One measured confidence in ability to overcome barriers and progress in career [11,12], with subjects rating the truth of each statement with an anchored seven-point ordinal scale from “completely false” to “completely true.” A related scale was developed to assess subjects’ perception of their potential for research success. Using an anchored six-point ordinal scale ranging from “not at all confident” to “completely confident,” subjects responded to statements about being successful in research, becoming a leader in research, securing research funding, and maintaining a research network.

#### Cultural awareness and appreciation of diversity

As secondary outcomes, the study sought to understand if Institute participants were more likely than their control counterparts to demonstrate improved cultural awareness and appreciation of diversity. This cross-cultural competence domain was assessed using three scales that assess: 1) cognitive empathy (the ability to comprehend other peoples’ experience), 2) valuing diversity, and 3) anti-sexism and anti-racism skills. The cognitive empathy measure we used consisted of a subset of items from a valid long instrument developed by Reniers and colleagues, the QCAE: A Questionnaire of Cognitive and Affective Empathy [24]. Drawing on items that best-suited workplace relationships, e.g., “I find it easy to put myself in someone else’s shoes,” the parsimonious cognitive empathy scale we adopted was constructed by shortening the QCAE cognitive empathy items from 19 to 6. To achieve a more differentiated distribution of responses, we adapted the original four-point agreement scale to an anchored seven-point ordinal scale ranging from “completely false” to “completely true.” Items included characteristics such as ability to predict how someone else might feel or what they might want to talk about, and whether someone is concealing their true emotions.

After conducting an extensive literature review, we wrote several new items to measure various aspects of cultural competence, since we were largely unable to find validated survey items tied to our hypothesized outcomes of greater cultural awareness and appreciation of diversity. The new survey items were pilot-tested, reviewed in conceptual groups, and examined by classical item analysis, factor analysis, and item response theory modeling using SAS/STAT Version 9.4 for Windows, 2006 (SAS Institute, Cary, NC) to arrive at a final set of scales and subscales. Our process resulted in the creation of a nine-item measure of valuing diversity that focused on attitudes related to recruitment, and diversifying workgroups and workplaces by gender, race, and ethnicity. For example, one item posed the statement, “I believe diverse teams produce better results than teams that are not diverse.” Subjects responded to each belief statement on an anchored six-point ordinal scale ranging from “very untrue” to “very true.” A third scale reflected subjects’ assessment of their ability to identify and effectively respond to incidents of gender, race and ethnicity inequity, e.g., “I can easily identify gender inequity.” These items used an anchored seven-point ordinal scale ranging from “completely false” to “completely true.”

#### Mentoring self-efficacy

We assessed subjects’ confidence in mentoring others using seven items adapted from our published and validated scale [10]: three items addressed professional goals – formulating them, identifying skills needed as well as specific plans to achieve their goals; two items concerned helping find the resources as well as a sponsor or champion to advance their work; one item was related to helping to define personal goals, and a final item assessed overall confidence in being an effective mentor.

### Data analysis

As described earlier, new survey items were examined by classical item analysis, factor analysis, and item response theory modeling to arrive at two cross-cultural measures: valuing diversity (attitudes and recruitment) and anti-sexism/anti-racism skills. The eight study outcome scales were tabulated and their psychometric properties assessed by item correlation and Cronbach alpha (Table 1).

In order to maintain the integrity of the stratified randomized design, all missing data were imputed. In cases of within-scale item missingness, for scales in which fewer than 50% of items were missing for a given respondent at a given time point, items were imputed in a single expected maximization imputation using all available items across all subjects at both time points in IBM SPSS Statistics Version 28.0 (SPSS, Inc., Chicago, IL) and scales were computed using all nonmissing and imputed items. In all other cases, including instances in which 50% or more of items in one scale were missing, 100 data sets were imputed via multiple imputation in Stata 17 (StataCorp, College Station, TX) using regression imputation based on all available scales and membership in the intervention or waitlist control condition.

Posttest scores were evaluated via regression. All models included the pretest or baseline version of the outcome variable and the three study design stratifiers as dummy variables: race and ethnicity, gender, and degree. All models were estimated using the MI estimate (for multiply imputed data) command in Stata.

## Results

The 40 subjects came from 27 medical schools in 16 states.

Because one member of the intervention group and one member of the control group withdrew and therefore had no posttest data, all of their posttest data were imputed. The response rate for participants who received the online surveys was 100%. We attribute the high response rate to the substantial benefit perceived by faculty participating in the intervention, a tuition subsidy, and additionally to frequent follow-up by the study’s external data collection center. The characteristics of the sample of 20 intervention and 20 waitlist control subjects at pretest are found in Table 2. For one outcome, two additional participants beyond the two who withdrew had missing data that was imputed during multiple imputations.

**Table 2.** Characteristics of 40 subjects participating in the C-Change group peer mentoring study

Characteristics	Intervention (*n* = 20)	Waitlist control (*n* = 20)
	Number (%)	Number (%)
Female	10 (50)	10 (50)
Race and ethnicity: URM*	10 (50)	10 (50)
Race and ethnicity by gender		
Non-URM* male	5 (25)	5 (25)
Non-URM female	5 (25)	5 (25)
URM male	5 (25)	5 (25)
URM female	5 (25)	5 (25)
Degree		
Ph.D.	9 (45)	10 (50)
M.D.	7 (35)	8 (40)
Both M.D. & Ph.D.	4 (20)	2 (10)
Rank		
Assistant professor	7 (35)	11 (55)
Associate professor	13 (65)	9 (45)
NIH research award		
K award recipients	7 (35)	12 (60)
R01 award recipients	10 (50)	6 (30)
Other research award^§^	1 (5)	2 (10)
Mean years in academic medicine in 2019 (SD)	9.6 (2.2)	8.6 (3.2)
Mean number of publications (SD)	32.4 (17.2)	29.4 (20.4)

* Non-underrepresented in medicine (Non-URM) and underrepresented in medicine (URM): Individuals from racial and ethnic groups that are adequately represented and have low representation, respectively, in the health-related sciences and STEM fields on a national basis, as designated by the National Institutes of Health and the National Science Foundation. Notice of NIH’s Interest in Diversity. National Institutes of Health. November 22, 2019. (https://grants.nih.gov/grants/guide/notice-files/NOT-OD-20-031.html). Accessed June 22, 2023.

§ Other research award includes major grants from professional organizations and foundations.

The results of imputed regression analyses are shown in Table 3. Primary outcomes: for the vitality outcome there was no significant intervention effect *(B* = 0.277; 95% CI: −0.158, 0.712; *D* = 0.309); however, the perceived change in vitality was significantly different between the intervention and control groups *(B* = 1.496; 95% CI: 0.191, 2.801*; D* = 1.473). For the second primary outcome, self-efficacy for career advancement was marginally higher in the intervention group compared to controls *(B* = 0.559; 95% CI: −0.001, 1.119; *D* = 0.406). Self-efficacy for research was higher in the intervention group *(B* = 0.607; 95% CI: 0.099, 1.115; *D* = 0.574).

**Table 3.** Estimated intervention coefficients (with 95% CIs, effect sizes (D), *T* values, and *P* values) from multiply imputed regression models comparing C-Change intervention and control subjects at Time 1^a^, controlling for Time 0^a^, gender, race and ethnicity, and degree

Outcome	Adjusted differences between groups at T1^a^		Effect size *(D)* ^*b*^	*T*-ratio	*P-v*alue
	Regression Coefficient *(B)*	95% CI			
*Primary outcomes*					
Vitality	0.277	−0.158, 0.712	0.309	1.31	0.20
Self-assessed change in vitality	1.496	0.191, 2.801	1.473	2.34	0.03
Self-efficacy: career advancement	0.559	−0.001, 1.119	0.406	2.04	0.05
Self-efficacy: research	0.607	0.099, 1.115	0.574	2.43	0.02
*Secondary outcomes*					
Value diversity: attitudes & recruitment	0.386	0.068, 0.703	0.460	2.48	0.02
Cognitive empathy	0.806	0.085, 1.526	0.846	2.28	0.03
Anti-sexism/anti-racism skills	0.763	0.201, 1.326	0.714	2.77	0.01
Self-efficacy: mentoring others	0.926	0.279, 1.572	1.138	2.94	0.007

a Timepoints Time^0^ and Time^1^ represent measurement prior to and following the intervention period.

b Effect size *D* calculated from regression coefficient for intervention effect representing adjusted standardized mean difference.

Secondary outcomes: with regard to valuing diversity, the intervention group was significantly higher than the control group at posttest *(B* = 0.386; 95% CI: 0.068, 0.703; *D* = 0.460). The intervention group also scored higher on cognitive empathy *(B* = 0.806; 95% CI: 0.085, 1.526; *D* = 0.846) than the controls; similarly, intervention outscored control on anti-sexism/anti-racism skills *(B* = 0.763; 95% CI: 0.201, 1.326; *D* = 0.714). Finally, the intervention group demonstrated improvement in self-efficacy for mentoring others as compared with the control group (*B* = 0.926; 95% CI: 0.279, 1.572; *D* = 1.138).

Using the widely accepted standard for effect sizes [27], where an effect size – representing a standardized mean difference between two groups in standard deviation units, or *D –* of .3 is considered “medium” and .5 is considered “large,” the statistically significant intervention effects noted in this study expressed as *D* are generally large (e.g., valuing diversity or self-efficacy in research), but often very large (e.g., perceived change in vitality or self-efficacy mentoring others). The smallest significant effect lies halfway between medium and large (self-efficacy in career advancement).

To assess any artifacts of multiple imputations – particularly imputing posttest data for each of two dropouts, one from treatment and one from control – we conducted a sensitivity analysis comparing the fully imputed results to complete cases analysis, imputed results omitting the two dropouts, and an imputation that moved the treatment dropout from treatment to control during imputation and back to treatment for analysis. All results were highly similar. Only the last of these showed any difference in statistical significance in which two results moved to marginal insignificance.

## Discussion

This randomized controlled study of a facilitated group peer mentoring intervention demonstrated medium to very large positive effects in improving perceived change in vitality and increasing self-efficacy in career advancement, research success, and mentoring others. The intervention also caused improvement in cross-cultural awareness as measured by cognitive empathy, valuing diversity attitudes and recruitment, and recognition of sexism and racism – with improved self-reported skills to address both.

This intervention shifts the widely used dyadic model of mentoring to a facilitated group strategy wherein peers mentor each other. Doing so eliminates common pitfalls of dyadic mentoring [28–32]. Further, group peer mentoring reduces the demands on senior faculty members, and the resources required to reimburse and train one-on-one senior mentors. The intervention was intentionally a culture change paradigm whereby Institute participants experienced learning in a transformed culture that contrasts with that usually experienced in medical schools where hierarchy, individualistic competition, and isolation are prevalent [33]. Meetings enacted characteristics of the culture we believe are necessary for medical schools to support relationship formation, align personal core values and career goals, support the humanity of faculty, and include the perspectives and skills of faculty who belong to groups that are underrepresented in medicine.

A systematic review reported the paucity of quality studies that support conclusions made about the effect of mentoring on career development in medical schools [34]. The benefits of mentoring programs for physicians-in-training or junior faculty have been described by a variety of qualitative and quantitative reports: for example, higher levels of self-efficacy in career and skill development [15,34,35], academic success (awards, grants, teaching/mentoring, publications) [8], career satisfaction [36], and scholarly activity and productivity [9,37,38,]. A few studies have focused on mentoring of research careers in academic medicine [9,39–41], including a survey of junior research awardees of NIH K08 and K23 grants, which mandate mentoring, where mentoring was significantly associated with career satisfaction [42].

By design, this mentoring intervention aims to foster personal growth, self-knowledge, development, and optimal learning in adults; creates a framework and environment for career guidance and personal awareness; provides ways to fully include women and persons from groups that are underrepresented in medicine and groups that experience marginalization in the larger society; minimizes problems of hierarchy and power differentials; and may reduce issues of gender, race, and ethnicity discordance. In addition to our own work [14,15,35,38], we note published descriptions of successful peer mentoring programs for junior faculty in the U.S. and overseas, conducted in single institutions, all of which cite the C-Change mentoring program [43–49]. Most of these programs targeted female faculty. We are not aware of rigorous studies testing the efficacy of facilitated group peer mentoring.

The intervention brings attention and training in cultural diversity into the mainstream of career development for all groups of faculty, rather than secluding it in programs specifically addressing the needs of homogeneous groups. The Institute emphasized the effect of the culture rather than implying the deficiencies of certain subgroups of faculty. In our experience, typically, neither faculty of color nor White faculty has previously engaged in such constructive cross-cultural conversations at a personal level with colleagues because cross-cultural conversations about differences are expected to be uncomfortable, and they are generally avoided in professional life.

To assess the longer-term effects given that the waitlist group will also be treated, we constituted an untreated, nonrandom control group through propensity score matching of subjects from the same sampling frame. Over the course of several years, we will follow and compare the two intervention groups with the nonintervention propensity score matched control group both on the dimensions measured in this study and on traditional markers of academic achievements, such as promotions, leadership positions, success obtaining research grants, publications, and retention in academic medicine and research.

### Limitations

First, the sample size was small and therefore the results are imprecise, even though most of the outcomes were statistically significant. In particular, the small sample size meant that there was inadequate statistical power to assess for different effects across demographic subgroups, even though the trends were all in the same direction.

Second, the sample might not be representative of the target population of early midcareer medical research faculty because the study relied on volunteers who sought enrollment in the Institute, a career development program with a substantial time and travel commitment. It is impossible to know either the direction or strength of volunteer bias on any of the variables studied. Those seeking to attend the Institute might on average be high achievers looking to further advance their careers, or they might be struggling with their career choices and hoping for a boost.

Third, the Institute used two highly-trained, experienced facilitators. It is uncertain how much of the positive intervention effect is attributable to the specific characteristics of the facilitators.

## Conclusions

This randomized trial, although small, demonstrates that a novel intervention consisting of facilitated group peer mentoring for early midcareer medical researchers can effect meaningful change in important dimensions and self-reported skills. The mentoring intervention successfully addresses the twin urgencies of sustaining faculty vitality and cross-cultural engagement and inclusion. Further research is required to replicate these results, clarify the C-Change Mentoring & Leadership Institute’s impact on various demographic groups, and assess whether the positive effects are sustained.
